# Wearable Devices in Elderly Chronic Disease Management: A Qualitative Study of Barriers and Facilitators

**DOI:** 10.1155/jonm/1278057

**Published:** 2025-11-28

**Authors:** Huichao Zhang, Jiayi Xu, Xinyu Lu, Mingmei Xu

**Affiliations:** ^1^The Second Hospital of Nanjing, Affiliated to Nanjing University of Chinese Medicine, Nanjing, Jiangsu, China; ^2^Suzhou Medical College of Soochow University, Soochow, Jiangsu, China; ^3^Nursing College, Nanjing University of Chinese Medicine, Nanjing, Jiangsu, China

**Keywords:** chronic disease management, elderly patients, healthcare providers, qualitative study, wearable health devices

## Abstract

**Aim:**

To explore the perceptions of elderly patients with chronic diseases and their healthcare providers on the use of wearable devices for home health monitoring, identifying barriers and facilitators to their effective adoption in China's rapidly aging population.

**Background:**

As China's population ages, managing chronic diseases among elderly patients has become increasingly complex. Wearable health monitoring devices offer a promising solution, enabling remote and continuous health tracking. However, the adoption of these devices remains limited, particularly among elderly patients. Understanding the perspectives of both patients and healthcare providers is crucial for optimizing wearable device use in chronic disease management.

**Design:**

A qualitative descriptive study.

**Method:**

From May 2023 to March 2024, semistructured interviews were conducted with 16 elderly patients and 11 healthcare providers in Nanjing, China. Participants were selected through purposive sampling. Data were analyzed using Colaizzi's 7-step method, generating key themes that address both the challenges and opportunities in adopting wearable health technologies.

**Findings:**

Seven major themes emerged: (1) Technology Acceptance and Motivation, (2) Changes in Social Support and Interaction, (3) Adjustments in Healthcare Work Modes, (4) Barriers and Risks in Technology Application, (5) Device Compliance and Knowledge Gaps, (6) Identifying Key Features for Quality Assessment of Wearable Devices, and (7) The Role of Customization and Adaptability. While the potential benefits of wearable devices for chronic disease management were widely recognized, concerns about complexity, cost, and data security were key obstacles.

**Conclusion:**

Wearable devices hold significant potential for improving the management of chronic diseases among elderly patients, yet multiple barriers hinder their widespread adoption. Addressing issues related to usability, privacy, and affordability, alongside providing education and policy support, will be critical to enhancing their integration into healthcare settings.

**Implications for the Profession and Patient Care:**

This study underscores the importance of creating targeted strategies to overcome the challenges in using wearable health devices among elderly patients. Healthcare providers and policymakers should focus on simplifying technology, enhancing patient education, and addressing privacy concerns to foster broader acceptance and use of these devices in chronic disease care.

**Impact:**

By promoting the adoption of wearable health technologies, healthcare systems can improve chronic disease management outcomes for elderly patients, reduce the burden on healthcare services, and support a more patient-centered approach to care.

**Patient or Public Contribution:**

Elderly patients with chronic diseases and their healthcare providers contributed valuable insights by sharing their experiences with wearable health monitoring devices, shaping the study's findings.

**Summary**

• What does this paper contribute to the wider global clinical community?
◦ This study provides insights into the barriers and facilitators affecting the adoption of wearable health technologies among elderly patients and their healthcare providers.
◦ The findings offer practical guidance for healthcare systems globally in addressing usability, privacy, and affordability challenges in integrating wearable devices into chronic disease management.
◦ The study's focus on an aging population highlights its relevance to countries facing similar demographic shifts, enabling broader application of its recommendations in global health settings.

## 1. Introduction

The rapid aging of populations globally, particularly in China, has introduced unprecedented challenges to healthcare systems, especially in the management of chronic diseases among elderly individuals [1]. By 2020, individuals aged 60 years or older comprised 18.7% of China's total population, a figure that continues to grow as birth rates decline [2, 3]. This demographic shift has placed immense strain on healthcare services, as the elderly not only require more frequent medical interventions but also face prolonged treatment durations due to chronic conditions such as hypertension, diabetes, and cardiovascular disease [4, 5]. Traditional in-person care models, which rely heavily on regular hospital visits and scheduled checkups, are increasingly inadequate in meeting the complex, long-term needs of elderly patients with chronic diseases [6].

In response to this emerging crisis, digital health technologies, particularly wearable devices, have been proposed as a solution to bridge the gap between healthcare demand and supply [7]. Wearable devices, capable of continuously monitoring physiological parameters such as heart rate, blood pressure, and sleep patterns, provide real-time data that enable healthcare providers to remotely track the health status of elderly patients [8, 9]. This technology not only offers the potential to improve the efficiency and accuracy of chronic disease management but also reduces the burden on healthcare facilities by minimizing the need for in-person visits [10]. Recent studies have highlighted the clinical benefits of wearable devices, noting their efficacy in early detection of health deterioration and prevention of disease complications [11, 12]. However, despite these promising advancements, the adoption and sustained use of wearable devices among elderly patients remain limited [13].

While much of the existing research on wearable devices focuses on their technical capabilities and clinical outcomes, far less attention has been paid to the perspectives of elderly patients and healthcare providers—the primary users and implementers of these technologies. Elderly patients, due to cognitive decline, limited technological literacy, and specific health concerns, face unique barriers to the effective use of wearable devices [14, 15]. Similarly, healthcare providers must adapt their workflows to integrate wearable-generated data into clinical practice, which presents challenges in terms of data management, resource allocation, and patient engagement [16]. Understanding the real-world factors that influence the acceptance and utilization of wearable devices by both patients and providers is critical to addressing these barriers and optimizing their implementation in elderly care settings.

Moreover, concerns surrounding data privacy, security, and economic accessibility further complicate the widespread use of wearable devices in healthcare [17]. For elderly patients, issues of data protection and trust in digital technologies are paramount, as many are unfamiliar with how their health data are collected, stored, and shared [18]. Healthcare providers, on the other hand, are tasked with ensuring that patient data are handled securely while simultaneously managing the logistical demands of monitoring and responding to real-time health information. Additionally, the cost of wearable devices remains a significant deterrent for many elderly patients, particularly in low- to middle-income settings [19]. Thus, economic barriers, coupled with privacy concerns, further limit the potential for wearable technology to be scaled across broader healthcare systems.

Given these multifaceted challenges, a gap persists in understanding the socioeconomic, technological, and psychological factors that shape the adoption of wearable devices among elderly patients and healthcare providers. Existing studies predominantly address technical aspects of the devices or their impact on clinical outcomes, without adequately considering the user experience or the broader systemic constraints that hinder their integration into everyday care practices. This study aims to explore the perceptions of elderly patients with chronic diseases and their healthcare providers regarding the home use of wearable devices for health monitoring. Specifically, this qualitative research seeks to identify the barriers and facilitators of wearable device adoption, offering insights that could inform future policy and practice in chronic disease management for aging populations.

## 2. Background

The use of wearable devices in health monitoring has gained significant momentum, particularly in chronic disease management. These devices provide continuous data on vital signs, such as heart rate and blood pressure, offering healthcare providers the ability to monitor patients remotely and intervene proactively. Wearable devices hold great potential to address the growing demand for healthcare services, particularly as populations age and chronic diseases become more prevalent. Numerous studies have documented the effectiveness of wearable devices in improving patient outcomes for conditions like hypertension, diabetes, and cardiovascular diseases. von Storch K et al. [20] found that wearable technology enhances patient engagement and self-management, while Lu et al. [21] demonstrated how wearables support healthier lifestyle choices, thus preventing the exacerbation of chronic conditions.

However, despite these advantages, the adoption of wearable devices among elderly patients remains low. Elderly individuals face distinct challenges, including reduced digital literacy, cognitive decline, and physical impairments that hinder their ability to operate wearable technologies. Studies, such as those by Lemus et al. [22], emphasize that anxiety about using new technology is common among older adults, contributing to their reluctance to adopt wearable devices.

To understand the factors influencing technology adoption, the technology acceptance model (TAM) provides a useful framework [23]. TAM posits that the acceptance of technology is driven primarily by two factors: perceived ease of use and perceived usefulness [24]. In the context of wearable devices, elderly patients may reject the technology if they perceive it as too complicated or not directly beneficial to their health. For elderly populations, perceived complexity and the lack of clear, immediate health benefits present significant barriers to the adoption of wearable devices. In addition to TAM, the health belief model (HBM) provides a complementary perspective on understanding health-related behaviors and technology adoption [25]. HBM suggests that individuals' health behaviors are influenced by their perceptions of vulnerability to illness, the seriousness of health risks, and the perceived benefits of taking action, such as using wearable devices [26]. According to Khodaveisi et al. [27], HBM posits that the higher the perceived threat of a health condition, the more likely an individual is to engage in preventive behaviors. In the case of elderly patients with chronic diseases, wearable devices may be more readily adopted if patients perceive a significant threat to their health and believe that the technology can effectively reduce this threat. However, barriers such as cost, privacy concerns, and data security may diminish their motivation to adopt these technologies, despite the perceived benefits.

Much of the existing literature has focused on younger populations who are more familiar with technology, leaving the unique challenges faced by elderly patients underexplored. Additionally, while frameworks like TAM and HBM offer valuable insights into the factors influencing technology adoption, there is limited research that applies these models specifically to elderly populations and wearable devices.

## 3. Methods

### 3.1. Study Design

This study employed a descriptive qualitative design, utilizing semistructured interviews to gather in-depth perspectives from older adults with chronic diseases and their healthcare providers [28]. The study adhered to the Consolidated Criteria for Reporting Qualitative Research (COREQ) guidelines [29].

### 3.2. Participants

Participants were recruited via purposive sampling from a tertiary hospital in Nanjing, China. Inclusion criteria for older patients were as follows: aged 65 years or older, diagnosed with at least one chronic condition, planned for hospital discharge, and having prior experience using health monitoring devices (e.g., health wristbands, rings) or expressing interest in the topic, and voluntary participation. Healthcare providers were required to have at least 3 years of experience in geriatric care. A total of 27 participants were interviewed, including 16 older patients and 11 healthcare providers.

### 3.3. Data Collection

Semistructured interviews were conducted using an interview guide developed based on the TAM and HBM frameworks, covering topics such as technology acceptance, perceived health risks, barriers to device use, and expectations. Interviews were conducted either face-to-face or online. Face-to-face interviews were held in a separate meeting room within the department, while online interviews were conducted using the WeChat video call function. No individuals other than the participant and the interviewer were present during the sessions. All participants consented to and were audio-recorded in Mandarin Chinese using a mobile phone recorder. Audio recordings were manually transcribed verbatim within 24 h after each interview by a researcher. To ensure consistency and accuracy, all interviews were conducted by the first author. She is a female nursing graduate student with systematic training in qualitative research methods and 3 years of clinical experience.

Initially, the researcher contacted eligible participants in person, introduced the study's purpose and content, invited them to participate, and scheduled the interview time and mode. Prior to the interview, participants provided verbal informed consent for audio recording. Demographic information was collected at the end of the interview. The interview guide was designed based on the TAM and HBM frameworks and was refined through group discussions and pretest interviews with two participants. The final interview guide (in Appendix A) included exploratory questions such as “Could you give an example?” and “Could you explain further?” for clarification.

Interview durations ranged from 20 to 70 min. Data collection and analysis occurred concurrently until no new information emerged, indicating data saturation [30]. Ultimately, no new codes were generated. The researcher took field notes during interviews, and no repeat interviews were conducted. Transcripts were returned to participants to confirm accuracy.

### 3.4. Data Analysis

This study adopted Colaizzi's seven-step analysis method combined with directed content analysis, using NVivo 14.0 software for text coding [31]. Prior to analysis, the researchers established an initial coding framework based on key constructs from the TAM and HBM, which served as the existing themes for this study. We remained open to the emergence of new themes throughout the analytical process. When transcript content did not fully align with these predefined themes, an iterative process of theme development and adjustment was employed through discussions within the research team.

The first author thoroughly familiarized herself with the interview transcripts by carefully reading all contents to develop a holistic understanding of the participants. Meaningful statements closely related to the research questions were identified and extracted, with semantic units relevant to the study objectives highlighted and coded. Recurring significant viewpoints were coded to form preliminary meaning units. The coding process involved labeling semantic units with descriptive codes that remained close to the original text and were minimally abstract or interpretive [32], thereby reducing the risk of omitting essential content. Similar codes were then clustered to form themes, theme groups, and categories, establishing logical connections. The intrinsic relationships between themes and participants were described in detail to ensure comprehensive interpretation, followed by an in-depth analysis of the interrelationships among elements. Codes were categorized into predefined or new themes based on similarities and differences. Finally, the findings were returned to participants for validation to ensure accuracy and reliability.

The coding team, consisting of one nurse with a bachelor's degree and two nursing graduate students, discussed and revised codes through meetings until consensus was reached. All authors had experience in nursing ethics or qualitative research. The first author translated representative quotations, codes, subthemes, and themes into English.

### 3.5. Trustworthiness

To enhance the trustworthiness of the study, several strategies were employed [33]. Participants with diverse roles and educational backgrounds were invited to enable multiperspective understanding of the research question. To ensure credibility, typical responses from interviews were directly quoted in the results, and feedback was sought from collaborating researchers, experts, and participants. All participants confirmed that the results aligned with their experiences. Dependability was achieved through open discussions within the team: Data analysis was independently conducted by two authors and verified by another to ensure reliability and rigor. Additionally, interview questions were reviewed by experts prior to the study and validated through a small-scale pilot test before formal interviews to ensure suitability and reliability for the target population. For transferability, the research context, participant selection, data collection, and analysis processes were described in detail, accompanied by rich examples to aid reader comprehension. Researchers continuously reflected on their positioning to improve objectivity and accuracy.

### 3.6. Ethical Considerations

This study was approved by the Ethics Review Committee of Nanjing Medical University (2022SR617), and informed consent was obtained from all participants. The study strictly followed the principles of the Declaration of Helsinki [34]. Participation was voluntary, and participants had the right to withdraw at any time. Data were treated confidentially and accessible only to the research team. None of the authors was directly involved in the care of the interviewees.

## 4. Results

All contacted healthcare providers and older adult patients participated in the study punctually. A total of 16 older patients were ultimately included, with an age range of 65–84 years. Additionally, 11 healthcare providers were enrolled, ranging in age from 29 to 47 years. The basic characteristics of the older patients are presented in Table 1, and those of the healthcare providers are summarized in Table 2.

During the data analysis process, 89 codes were generated and categorized into seven themes (Table 3): Technology Acceptance and Motivation, Changes in Social Support and Interaction, Adjustments in Healthcare Work Modes, Barriers and Risks in Technology Application, Device Compliance and Knowledge Gaps, Identifying Key Features for Quality Assessment of Wearable Devices, and The Role of Customization and Adaptability.

### 4.1. Technology Acceptance and Motivation

Elderly patients' acceptance of wearable devices appeared multifaceted, shaped by both the perceived utility and ease of use, as framed by the TAM. While many participants highlighted the perceived usefulness of these devices in managing chronic conditions, the complexity of the technology presented notable barriers to widespread adoption. Participant P5 (aged 72 years, diabetes) shared, *“The device lets me track my blood pressure. It gives me peace of mind knowing that I can monitor my health from home.”* This acknowledgment of the technology's benefit mirrors TAM's perceived usefulness, as patients recognized the potential for better health management.

However, technological literacy proved to be a significant determinant of engagement. Older patients, or those with less prior experience with technology, reported difficulties in navigating the device's features. Participant P12 (aged 78 years, hypertension) expressed frustration: *“There are so many buttons and features that I just don't know where to start. I wish it were simpler.”* This aligns with TAM's perceived ease of use, suggesting that for many patients, the complexity of the device hindered its perceived utility, regardless of the potential health benefits. Similarly, healthcare providers observed these challenges, with Nurse N3 noting *“For many elderly patients, the technology is more of a burden than a help unless we provide constant support.”*

### 4.2. Changes in Social Support and Interaction

The introduction of wearable devices into daily life subtly transformed patients' interactions with their families, offering both practical and emotional shifts. This transformation aligns with the HBM, particularly in how elderly patients weighed the benefits and barriers of using such technology for health monitoring. Participant P1 (aged 69 years) commented, *“My daughter now checks my health through her phone. It makes her feel more secure, and I feel more connected with her, even when she's not around.”* This enhanced sense of connectedness reflects the perceived benefits of wearable devices, allowing family members to remain involved in health monitoring from a distance.

However, not all participants viewed this change positively. Several elderly patients voiced a sense of emotional disconnect despite the practical advantages. Participant P16 (aged 75 years) shared, *“Even though my family can see my health data, it's not the same as having them here with me. The technology helps, but it doesn't replace the warmth of having them by my side.”* This sentiment captures the limitations of wearable technology in addressing the broader emotional needs of elderly patients. While the devices provide a functional form of support, they do not mitigate the perceived barriers that come from a lack of direct, emotional engagement, highlighting the nuanced role of technology in social interactions within families.

### 4.3. Adjustments in Healthcare Work Modes

Healthcare providers largely endorsed the utility of wearable devices, particularly in enabling more effective chronic disease management through continuous monitoring. This reflects TAM's perceived usefulness, as providers recognized the potential of these technologies to enhance patient care. Doctor N6 stated, *“Having access to real-time health data can help us intervene earlier, potentially preventing complications before they escalate.”* The value of continuous data streams was particularly emphasized in cases where early detection of issues could improve patient outcomes.

Nevertheless, providers also pointed to significant challenges in integrating this technology into existing healthcare workflows. Nurse N9 remarked, *“We're flooded with data, but without the infrastructure to process it efficiently, the information becomes overwhelming rather than helpful.”* This observation highlights TAM's perceived ease of use from the perspective of healthcare professionals: While the devices offer valuable information, the lack of systems to manage these data effectively undermines their practical utility. The balance between perceived usefulness and operational integration thus becomes a critical factor in determining the true impact of wearable devices on healthcare practices.

### 4.4. Barriers and Risks in Technology Application

Concerns surrounding privacy and data security were pervasive among elderly patients, reflecting the perceived barriers component of the HBM. Many participants expressed anxiety about the potential misuse of their health data, with Participant P14 (aged 77 years) articulating *“I'm afraid that someone else could access my information. I don't really understand how it's protected, and that worries me.”* This fear of vulnerability reflects the perceived susceptibility within HBM, where the uncertainty surrounding data protection limits the patient's confidence in using the technology.

Healthcare providers echoed these concerns, particularly regarding the technical risks associated with wearable devices. Nurse N1 highlighted, *“There's always the risk of system failures or data breaches, and that could delay treatments if we're relying too much on these devices.”* The dual concern from both patients and providers underscores a critical challenge: While wearable devices offer clear perceived benefits in health monitoring, the unresolved issues around privacy and security continue to act as significant barriers to broader adoption, particularly among more vulnerable populations like the elderly.

### 4.5. Device Compliance and Knowledge Gaps

Device compliance was closely tied to patients' understanding of how to use the technology, with TAM's perceived ease of use emerging as a central theme. Participant P10 (aged 74 years) noted, *“I sometimes get stuck trying to connect the device to my phone. When it doesn't work, I feel frustrated and don't know what to do.”* This sentiment captures the broader challenges elderly patients face in navigating wearable devices, with many lacking the necessary digital literacy to operate them smoothly.

In response, healthcare providers emphasized the importance of comprehensive and ongoing training. Nurse N8 commented, *“It's not enough to just set up the device for them. We need to give continuous support and offer easy-to-understand guidance to help them troubleshoot.”* This view aligns with both TAM and HBM, where proper education and support reduce perceived barriers and improve the perceived ease of use. Without sustained guidance, patients may struggle to engage fully with the technology, reducing its overall effectiveness in managing chronic conditions.

### 4.6. Identifying Key Features for Quality Assessment of Wearable Devices

In addition to the challenges surrounding usability and compliance, several participants identified specific features they considered critical when evaluating the quality of wearable devices. These insights can inform the development of a targeted evaluation tool, which prioritizes the practical needs of elderly users.

Participant P7 (aged 71 years) emphasized simplicity, stating *“The fewer buttons the better. I don't need all the fancy features, just something easy to read and understand.”* This highlights the need for a streamlined user interface, where ease of use is paramount for elderly patients. Participant P9 (aged 76 years) noted, *“It's important that the device doesn't need constant charging. I forget to charge things, so it needs to last long.”* This practical concern about battery life points to a critical feature in evaluating device reliability from an elderly perspective.

Furthermore, safety and privacy remained top concerns. Participant P14 remarked, *“I'd like to know more about how my information is protected. If I could see clear instructions on that, I'd feel more comfortable.”* This suggests that transparency around data security should be a key component in any quality evaluation framework, ensuring that elderly users have confidence in the protection of their health data.

### 4.7. The Role of Customization and Adaptability

Participants also expressed the desire for devices that could be customized according to their specific health needs. Participant P3 (aged 70 years) said, *“My health concerns are different from others. I need something that focuses on my heart, but I don't care about steps or sleep tracking.”* This sentiment underscores the importance of device adaptability and personalization, where the quality of the device is judged not just by its general functionality, but by its ability to cater to individual health priorities. This could be an important dimension in the development of a comprehensive evaluation tool for home monitoring devices.

## 5. Discussion

This study provides critical insights into the perceptions of elderly patients with chronic diseases and their healthcare providers regarding the use of wearable devices for home health monitoring. While the potential benefits of wearable technology in chronic disease management are widely acknowledged, several persistent challenges must be addressed to facilitate broader adoption. These challenges, which encompass technical, privacy, economic, and social dimensions, align with and extend findings in existing literature.

Another critical concern is the privacy and security of patient data. This issue emerged prominently in both patient and provider interviews, underscoring the need for stronger safeguards in digital health technologies. While measures such as encryption and regulatory compliance (e.g., HIPAA) are increasingly common, they do not always alleviate user concerns, particularly among elderly populations unfamiliar with digital privacy mechanisms [35]. Existing research has identified similar gaps in user understanding and trust, suggesting that addressing these issues requires more than technical solutions [36]. In addition to enhancing data protection, transparent communication about how data are used, stored, and protected is essential to building user trust and promoting the long-term adoption of wearable devices in healthcare settings.

Economic considerations also play a pivotal role in the adoption of wearable technologies. Many elderly participants in this study expressed concerns regarding the affordability of these devices, a finding corroborated by prior studies. Although wearable devices may reduce long-term healthcare costs by minimizing the need for frequent in-person consultations and preventing complications, their initial purchase and maintenance costs can be prohibitive [37]. Policymakers should consider financial support mechanisms, such as subsidies or insurance coverage, to make these technologies more accessible to low-income elderly populations [38, 39]. In parallel, incentivizing healthcare providers to adopt wearable technology in routine care through policy reforms or financial rewards could further encourage the integration of these devices into chronic disease management frameworks [40].

Usability, particularly for elderly users, remains another key challenge. The findings of this study reinforce the conclusions of previous research, which has consistently pointed to the need for simpler, more intuitive device designs. Many participants reported difficulties operating wearable devices, which detracted from their perceived usefulness. Design improvements, such as incorporating larger displays, voice activation, and minimal setup procedures, could significantly enhance the user experience. Simplifying these devices without compromising functionality will be essential to ensuring broader user acceptance, particularly among older populations who may struggle with more complex technology [41]. The data in this research point to several critical factors—usability, battery life, data security, and adaptability—which must be prioritized when assessing the effectiveness of these devices in real-world settings. As the study moves forward in developing a quality evaluation tool, it becomes apparent that a user-centered design approach should be adopted. The future tool must incorporate metrics that reflect the simplicity and ease of use highlighted by participants, ensuring that the devices can be easily operated by users with varying levels of technological literacy. Additionally, battery life and device reliability are nonnegotiable factors that directly impact the device's usefulness in daily life, particularly for elderly individuals who may forget or find it difficult to charge devices frequently.

Finally, education and training emerged as critical factors influencing the successful use of wearable devices. Both patients and healthcare providers highlighted the need for comprehensive training programs to ensure that elderly patients can effectively use these devices. Consistent with the findings of earlier studies [42, 43], this research emphasizes that ongoing education, beyond initial device setup, is crucial to maintaining adherence. Healthcare providers should be equipped to offer structured training sessions, follow-up support, and user-friendly instructional materials that are specifically tailored to the cognitive and technical capabilities of elderly patients. Such interventions could significantly improve device adherence and maximize the health benefits associated with continuous monitoring [44].

### 5.1. Future Research Directions

In line with these findings, future studies should focus on further refining and testing the proposed quality evaluation tool, ensuring that it addresses the diverse and specific needs of elderly users. This tool could be tested in broader populations and adjusted based on direct feedback from elderly participants, healthcare providers, and caregivers. Additionally, longitudinal studies could explore how elderly users' perceptions of wearable devices evolve over time as they become more familiar with the technology and how this affects the criteria for evaluating device quality. The findings of this study may provide insights for other regions or healthcare systems, particularly in the adoption of digital health technologies. We discuss the potential applicability of these findings in different cultural and healthcare settings.

## 6. Conclusion

Wearable devices hold great promise for improving the health management of elderly patients with chronic diseases. However, challenges related to device usability, privacy, and cost must be addressed to ensure the successful implementation of this technology in home healthcare settings. Future efforts should focus on simplifying device interfaces, securing data transmission, and providing policy support to promote broader adoption of wearable technology. This study offers valuable insights for future research and the development of interventions to optimize the use of wearable devices in chronic disease management.

## Figures and Tables

**Table 1:** Characteristics of the participants (elderly patients).

No.	Gender	Age (years)	Educational level	Main chronic diseases	Previous devices	Common devices
P1	Female	69	Senior high school	Hypertension	Wearable ECG patch, smartwatch-based blood pressure monitor	Smart wristband
P2	Female	65	Senior high school	Type 2 diabetes mellitus	Continuous glucose monitoring (CGM) system	Smart wristband
P3	Male	70	Associate degree	Hypertension	Wearable ECG patch	Smart wristband
P4	Female	77	Primary school	Hypertension	—	Smart wristband
P5	Male	72	Senior high school	Type 2 diabetes mellitus, hypertension	—	Smartwatch-based blood pressure monitor
P6	Male	68	Associate degree	Hypertension	—	Smart wristband
P7	Male	71	Primary school	Type 2 diabetes mellitus, hypertension	Smart ring, continuous glucose monitoring (CGM) system, smartwatch-based blood pressure monitor	Smart wristband
P8	Female	77	Secondary technical school	Type 2 diabetes mellitus, hyperlipidemia	Continuous glucose monitoring (CGM) system	Smart wristband
P9	Female	76	Primary school	Hypertension	—	Smart wristband
P10	Female	74	Secondary technical school	Hypertension	—	Smart wristband
P11	Female	80	Primary school	Hypertension	—	Smart wristband
P12	Male	78	Senior high school	Hypertension	—	Smart wristband
P13	Female	84	Senior high school	Hypertension, Type 2 diabetes mellitus	Smart ring, continuous glucose monitoring (CGM) system	Smart wristband
P14	Female	77	Bachelor's	Hypertension, cor pulmonale	Smartwatch-based blood pressure monitor, patch-type ECG monitor	Smart wristband
P15	Male	70	Bachelor's	Hyperuricemia	—	Smart wristband
P16	Male	67	Primary school	Hypertension	—	Smart wristband

*Note:* Previous devices are defined as wearable health monitoring devices that participants had prior experience with, regardless of frequency of use.

Common devices are defined as those used by participants on a regular basis in their daily lives.

**Table 2:** Characteristics of the participant (healthcare providers).

No.	Gender	Age (years)	Educational level	Work experience (years)	Ward	Profession
N1	Female	33	Bachelor's	10	Geriatric Hematology Department	Nurse
N2	Female	41	Master's	14	Geriatric Hematology Department	Head nurse
N3	Female	45	Bachelor's	25	Geriatric Respiratory Department	Head nurse
N4	Male	32	Master's	5	Geriatric Respiratory Department	Doctor
N5	Female	47	Master's	19	Geriatric Hematology Department	Doctor
N6	Female	40	Associate degree	21	Geriatric Respiratory Department	Nurse
N7	Female	38	Bachelor's	14	Geriatric Respiratory Department	Nurse
N8	Male	30	Bachelor's	4	Geriatric Intensive Care Unit	Nurse
N9	Female	29	Associate degree	7	Geriatric Intensive Care Unit	Nurse
N10	Female	34	Bachelor's	11	Geriatric Gastroenterology Department	Nurse
N11	Female	33	Bachelor's	10	Geriatric Gastroenterology Department	Nurse

**Table 3:** Theme summary and representative participant responses.

Theme	Representative participant responses
1. Technology Acceptance and Motivation	Participant P5 (aged 72 years, diabetes): “The device lets me track my blood pressure. It gives me peace of mind knowing that I can monitor my health from home.”
	Participant P12 (aged 78 years, hypertension): “There are so many buttons and features that I just don't know where to start. I wish it were simpler”
	Healthcare Provider N3: “For many elderly patients, the technology is more of a burden than a help unless we provide constant support”

2. Changes in Social Support and Interaction	Participant P1 (aged 69 years): “My daughter now checks my health through her phone. It makes her feel more secure, and I feel more connected with her, even when she's not around.”
	Participant P16 (aged 75 years): “Even though my family can see my health data, it's not the same as having them here with me.”

3. Adjustments in Healthcare Work Modes	Healthcare Provider N6: “Having access to real-time health data can help us intervene earlier, potentially preventing complications before they escalate.”
	Nurse N9: “We're flooded with data, but without the infrastructure to process it efficiently, the information becomes overwhelming rather than helpful.”

4. Barriers and Risks in Technology Application	Participant P14 (aged 77 years): “I'm afraid that someone else could access my information. I don't really understand how it's protected, and that worries me.”
	Healthcare Provider N1: “There's always the risk of system failures or data breaches, and that could delay treatments if we're relying too much on these devices.”

5. Device Compliance and Knowledge Gaps	Participant P10 (aged 74 years): “I sometimes get stuck trying to connect the device to my phone. When it doesn't work, I feel frustrated and don't know what to do.”
	Nurse N8: “It's not enough to just set up the device for them. We need to give continuous support and offer easy-to-understand guidance to help them troubleshoot.”

6. Identifying Key Features for Quality Assessment of Wearable Devices	Participant P7 (aged 71 years): “The fewer buttons the better. I don't need all the fancy features, just something easy to read and understand.”
	Participant P9 (aged 76 years): “It's important that the device doesn't need constant charging. I forget to charge things, so it needs to last long.”
	Participant P14: “I'd like to know more about how my information is protected. If I could see clear instructions on that, I'd feel more comfortable.”

7. The Role of Customization and Adaptability	Participant P3 (aged 70 years): “My health concerns are different from others. I need something that focuses on my heart, but I don't care about steps or sleep tracking.”
	Participant P3: “I need something that focuses on my heart, but I don't care about steps or sleep tracking.”

## Data Availability

The data that support the findings of this study are available from the corresponding author upon reasonable request.

## Supporting Information

Supporting Information. Interview Protocol.
