# Supplementary material for: Wearable Devices in Elderly Chronic Disease Management: A Qualitative Study of Barriers and Facilitators
Source: J Nurs Manag. 2025 Nov 28;2025:1278057. doi: 10.1155/jonm/1278057 (PMC12680462; doi:10.1155/jonm/1278057)
Supplement: Supporting Information — Additional supporting information can be found online in the Supporting Information section. [file 1278057.f1.docx]

**Interview Protocol**

**Part 1: Introduction and Background**

1. First, could you please briefly describe your general health condition since your recent discharge and your daily health management routine?
2. What health concerns are you most focused on in your current life? How severe do you perceive the potential consequences of these issues to be if not managed properly?

**Part 2: Device Usage and Perceptions**

1. What wearable health monitoring device(s) are you currently using (or have used)?

2. What was the main reason or cue that prompted you to start using it/them?

3. What benefits do you see in using these devices for managing your health?

4. What difficulties have you encountered or what do you find not useful or cumbersome about using them?

**Part 3: Needs, Concerns, and Expectations**

1. Regarding post-discharge health monitoring at home, what concerns or problems do you most hope the devices can help you address? To what extent do you feel the current devices meet these needs?
2. What are your primary concerns when using these devices?
3. How confident are you in your ability to use these devices correctly and understand the data they provide?
4. To better support your health management at home, what improvements or new features would you most like to see in future wearable devices?

**Part 4: Closing**

1. Is there anything we have discussed that you would like to add to or emphasize?
2. Thank you for taking the time to share your experiences and insights. This has been very helpful for our research.
